# Supplementary material for: The effects of clays on bacterial community composition during arthropod decay
Source: Swiss J Palaeontol. 2024 Jul 10;143(1):26. doi: 10.1186/s13358-024-00324-7 (PMC11236854; doi:10.1186/s13358-024-00324-7)
Supplement: Supplementary file 1 — Additional file 1. [file 13358_2024_324_MOESM1_ESM.docx]

**SUPPLEMENTARY MATERIALS**

**The effects of clays on bacterial community composition during arthropod decay**

Nora Corthésy^1*^, Farid Saleh^1*^, Camille Thomas^2^, Jonathan B. Antcliffe^1^, and Allison C. Daley^1^

^1^Institute of Earth Sciences, University of Lausanne, Géopolis, CH-1015 Lausanne, Switzerland

^2^Institute of Geological Sciences, Oeschger Centre for Climate Research, University of Bern, Baltzerstrasse 1+3, 3012 Bern, Switzerland

Corresponding authors: N. Corthésy ([nora.corthesy@unil.ch](mailto:nora.corthesy@unil.ch))

F. Saleh ([farid.nassim.saleh@gmail.com](mailto:farid.nassim.saleh@gmail.com))

**Morphological observations of decaying shrimps and quantification of the decay stages**

**Table S1.** Description of the seven decay stages (numbered from 0 to 6) of shrimps to quantify the decomposition.

| *Decay stage* | *Carapace* | *Appendages* | *Eyes* |
| --- | --- | --- | --- |
| 0 | Transparent | Transparent | Pigmented |
| 1 | Getting opaque | Getting opaque | Getting black |
| 2 | Formation of white biofilm or black film | Formation of with biofilm or black film | Completely black |
| 3 | Cuticle detaching from internal organs | Disarticulated | Detaching |
| 4 | Cuticle detached, exposure of gills and internal organs | Detached | Detached |
| 5 | Broken, split in pieces, internal organs gone | Broken | Degrading |
| 6 | Completely degraded | Completely degraded | Completely degraded |


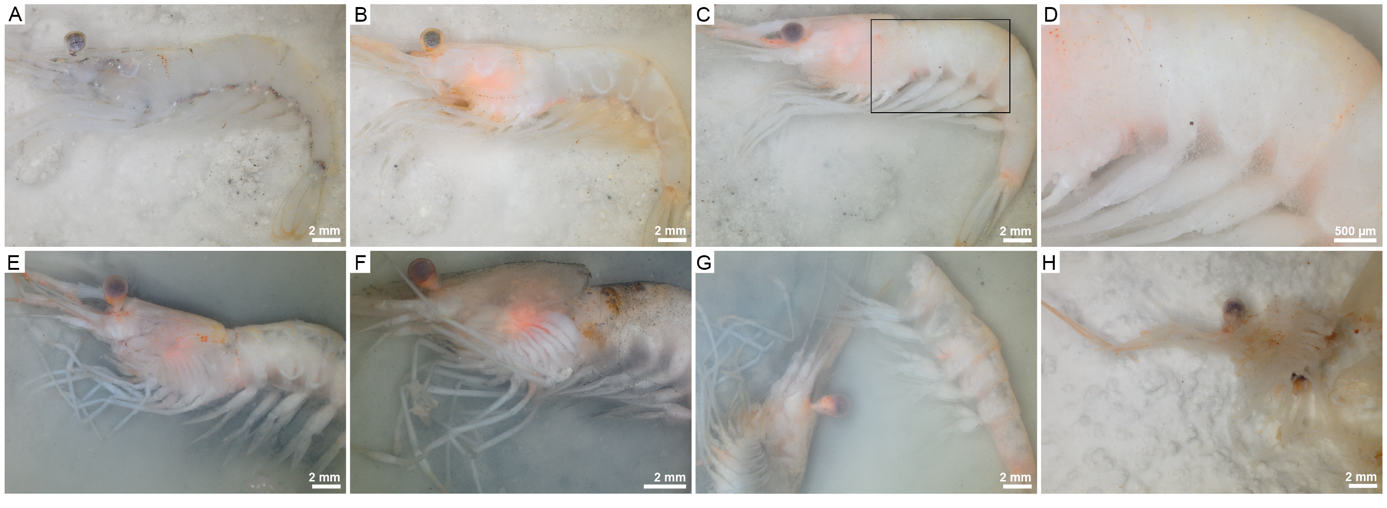


**Figure S1.** Example of the seven decay stages. (A) Decay stage 0 depicts an intact shrimp post-euthanasia. (B) Decay stage 1 is assigned when the shrimp undergoes color changes and becomes opaque. (C) Decay stage 2 is designated when white biofilms appear on the water surface and around the cuticle, as indicated in the rectangle. (D) Zoom in on the framed area of C. (E) Decay stage 3 is given when the cuticle begins to detach from the cephalothorax and abdomen, with the disarticulation of appendages. (F) Decay stage 4 involves the detached cuticle, exposing gills and internal organs, along with appendages detaching from the body. (G) Decay stage 5 signifies the separation of the cephalothorax from the abdomen, accompanied by highly degraded tissues. (H) Decay stage 6 represents complete degradation of the shrimp, with only a few remaining tissue fragments.

**Raw data and processed of the microbiome analyses can be found on the following link** (<https://doi.org/10.17605/osf.io/K6DHG>).

**Detailed statistical analyses**

**Table S2.** ANOVA comparing bacterial relative abundances according to clay minerals and cell walls (gram-positive/gram-negative), of the three clays.

|  | *DF* | *Sum square* | *Mean square* | *F-value* | *p-value* |
| --- | --- | --- | --- | --- | --- |
| Clay | 2 | 4 $\cdot$ 10^-5^ | 2 $\cdot$ 10^-5^ | 5 $\cdot$ 10^-4^ | 0.999 |
| Gram | 1 | 0.404 | 0.404 | 9.083 | 0.011 |
| Clay*Gram | 2 | 0.695 | 0.348 | 7.804 | 0.007 |
| Residuals | 12 | 0.534 | 0.045 |  |  |

**Table S3.** Contrast analyses to assess whether the proportions of gram-positive/gram-negative bacteria are influenced by the three different clays.

|  | *Estimate* | *Standard Error* | *t-ratio* | *p-value* | |
| --- | --- | --- | --- | --- | --- |
| **Clay = Bentonite** |  |  |  |  | |
| Negative – Positive | 0.028 | 0.172 | 0.161 | 0.875 | |
| **Clay = Kaolinite** |  |  |  |  | |
| Negative – Positive | 0.856 | 0.172 | 4.966 | 0.0003 | |
| **Clay = Montmorillonite** |  |  |  |  | |
| Negative – Positive | 0.016 | 0.172 | 0.093 | 0.927 | |
| **Gram = Negative** |  |  |  |  |  |
| Bentonite – Kaolinite | -0.413 | 0.172 | -2.396 | 0.080 |  |
| Bentonite – Montmorillonite | 0.003 | 0.172 | 0.019 | 0.999 | |
| Kaolinite – Montmorillonite | 0.416 | 0.172 | 2.415 | 0.078 | |
| **Gram = Positive** |  |  |  |  |  |
| Bentonite – Kaolinite | 0.415 | 0.172 | 2.408 | 0.079 |  |
| Bentonite – Montmorillonite | -0.008 | 0.172 | -0.049 | 0.999 | |
| Kaolinite – Montmorillonite | -0.423 | 0.172 | -2.458 | 0.072 | |

**Table S4.** ANOVA comparing bacterial relative abundances according to clay minerals and cell walls (gram-positive/gram-negative), when comparing kaolinite with bentonite and montmorillonite combined.

|  | *DF* | *Sum square* | *Mean square* | *F-value* | *p-value* |
| --- | --- | --- | --- | --- | --- |
| Clay | 1 | 2 $\cdot$ 10^-5^ | 2 $\cdot$ 10^-5^ | 6 $\cdot$ 10^-4^ | 0.981 |
| Gram | 1 | 0.404 | 0.404 | 10.594 | 0.006 |
| Clay*Gram | 1 | 0.695 | 0.695 | 18.203 | 0.001 |
| Residuals | 14 | 0.534 | 0.038 |  |  |

**Table S5.** Contrast analyses to assess whether the proportions of gram-positive/gram-negative bacteria are influenced by the different clays, when comparing kaolinite with bentonite and montmorillonite combined.

|  | *Estimate* | *Standard Error* | *t-ratio* | *p-value* |
| --- | --- | --- | --- | --- |
| **Clay = Kaolinite** |  |  |  |  |
| Negative – Positive | 0.856 | 0.160 | 5.363 | 0.0001 |
| **Clay = Other clays** |  |  |  |  |
| Negative – Positive | 0.022 | 0.113 | 0.194 | 0.849 |
| **Gram = Negative** |  |  |  |  |
| Kaolinite – Other Clays | 0.414 | 0.138 | 2.999 | 0.0096 |
| **Gram = Positive** |  |  |  |  |
| Kaolinite – Other Clays | -0.419 | 0.138 | -3.034 | 0.0089 |
